# The use of three-dimensional cephalometric references in dentoskeletal symmetry diagnosis

**DOI:** 10.1590/2176-9451.19.6.078-085.oar

**Published:** 2014

**Authors:** Olavo Cesar Lyra Porto, Jairo Curado de Freitas, Ana Helena Gonçalves de Alencar, Carlos Estrela

**Affiliations:** 1 Federal University of Goiás, PhD resident in Health Sciences, Federal University of Goiás (UFG); 2 Brazilian Dental Association, Department of Orthodontics, Professor, Department of Orthodontics, Brazilian Dental Association (ABOR); 3 UFG, Department of Endodontics, Professor, Department of Endodontics, UFG; 4 UFG, Department of Endodontics, Full professor, Department of Endodontics, UFG

**Keywords:** Facial asymmetry, Three-dimensional imaging, Cone-beam computed tomography

## Abstract

**OBJECTIVE:**

The aim of this study is to assess dentoskeletal symmetry in cone-beam computed tomography (CBCT) scans of Brazilian individuals with Angle Class I malocclusion.

**MATERIAL:**

A total of 47 patients (22 females and 25 males) aged between 11 and 16 years old (14 years) seen in a private radiology service (CIRO, Goiânia, GO, Brazil) were assessed. All CBCT scans were obtained from January, 2009 to December, 2010. Cephalometric measurements were taken by multiplanar reconstruction (axial, coronal and sagittal) using Vista Dent3DPro 2.0 (Dentsply GAC, New York, USA). Minimum, maximum, mean and standard deviation values were arranged in tables, and Student t-test was used to determine statistical significance (P < 0.05).

**RESULTS:**

Data were homogeneous, and differences between the right and left sides were not significant.

**CONCLUSIONS:**

Cephalometric measurements of Brazilian individuals with Angle Class I malocclusion can be used to establish facial symmetry and three-dimensional standard references which might be useful for orthodontic and surgical planning.

## Abstract (pt)

**OBJETIVO:**

o objetivo deste estudo é avaliar a simetria dentoesqueléticas em imagens de tomografia computadorizada de feixe cônico (TCFC) de indivíduos brasileiros com má oclusão Classe I de Angle.

**MÉTODOS:**

quarenta e sete pacientes (22 meninas e 25 meninos), com idades entre 11 e 16 anos (14 anos, em média), foram atendidos em um serviço de radiologia privado. Todas as imagens de TCFC foram adquiridas a partir de janeiro de 2009 a dezembro de 2010. Medições cefalométricas foram realizadas por reconstruções multiplanares (axial, coronal e sagital) usando o VistaDent 3D Pro 2.0 ( Dentsply GAC, Nova Iorque, EUA). O desvio-padrão mínimo, máximo e a média foram descritos em tabelas, e o teste *t* de Student foi utilizado para definir significância estatística (p < 0,05).

**RESULTADOS:**

os dados foram homogêneos e as diferenças entre os lados direito e esquerdo não foram significativas.

**CONCLUSÕES:**

as medidas cefalométricas de indivíduos brasileiros com má oclusão Classe I de Angle podem ser usadas para definir a simetria facial e referências de padrão tridimensional, que podem ser úteis para o planejamento ortodôntico e cirúrgico.

## INTRODUCTION

Assessing skeletal asymmetry by means of cephalometric and panoramic radiograph of individuals in need of orthodontic treatment is an ongoing challenge that requires attention. Knowledge about craniofacial growth and growth direction, skeletal anatomy, tooth position, tooth relationship with bone structures, and facial profile is essential for accurate treatment planning.^1^

Cephalometry focuses on linear and angular dimensions established by bone, teeth and face measurements; and cephalometric findings aid diagnosis and help to establish treatment strategies.

Dentists use lateral cephalogram to establish the cephalometric references of normal individuals with a balanced face.^2^^,^ 3 Despite potential limitations such as image distortion and superposition, posteroanterior radiograph is useful for other types of assessment. Nevertheless, it is considered reliable for surgical and orthodontic planning.^4^

Inaccurate image reading may be associated with superposition of anatomical structures and increased radiographic image distortion. Furthermore, correct management of patients during image acquisition is a risk factor that may affect quality. Two-dimensional radiographs are limited and might affect treatment planning and results negatively.^4^^,^ 5 ^,^ 6

The use of cone-beam computed tomography (CBCT) in Dentistry has raised several possibilities for planning, treatment and follow-up in a number of specialties.^7^^-^ 21 Farman and Scarfe^16^ reported that several CBCT systems may be used to obtain reconstructions similar to conventional cephalometric scans. According to these authors,^16^ CBCT diagnostic precision and efficacy may be compared to conventional cephalometric imaging. Additionally, they also state that evidence-based selection criteria should be developed for CBCT use in Orthodontics. 

Cephalometric analysis has been used to assess linear and angular measurements of hard and soft tissues of the craniofacial complex, while CBCT scans have been helpful in assessing facial asymmetry.^24^ New facial examination models may be developed by combining the use of conventional cephalometric references and three-dimensional CBCT scans.^25^^,^ 26 This study assessed dentoskeletal symmetry of Angle Class I patients by means of three-dimensional scans.

## MATERIAL AND METHODS

## Sample selection

Facial symmetry of a group of patients was determined and resulted in a clinically symmetrical sample. After that, three-dimensional scans of 47 patients (22 females and 25 males) aged between 11 and 16 years old (14 years) were retrieved and further assessed. The following inclusion criteria were applied: Angle Class I malocclusion, crowding, absence of dental caries and apical or marginal periodontitis. Exclusion criteria were: Angle Class II or III malocclusion, absence of teeth, traumatic bone and tooth injury, and previous orthodontic treatment. This study was approved by the local Institutional Review Board (Federal University of Goiás, Brazil, # 296/2011).

## Method used to determine facial symmetry

Patients' digital frontal facial photographs were assessed by three specialists in Orthodontics. Facial symmetry was determined according to visual inspection and facial photographs. Clinically symmetrical patients were selected for cephalometric measurements.

## Image acquisition method

CBCT scans were acquired in a private radiology clinic (CIRO, Goiânia, GO, Brazil) using an i-CAT scanner (Imaging Sciences International, Hatfield, PA, USA). Volumes were reconstructed according to the following exposure settings: 0.25-mm resolution, isometric voxel, 120 kVp, tube voltage, 3.8 mA current, exposure time of 40 seconds and field of view of 13 cm. Images were acquired at 14-bit grey scale at a focal distance of 0.5 mm and 360^o^ rotation.

Images were assessed by Xoran 3.1.62 software (Xoran Technologies, Ann Arbor, USA) in a workstation Intel Core(r) 2 Duo 1.86 Ghz-6300 processor (Intel Corporation, Santa Clara, USA), NVIDIA GeForce 6200 turbo cache video card (NVIDIA Corporation, Santa Clara, USA), EIZO - S2000 FlexScan monitor (1600 x 1200 pixels resolution) and Microsoft Windows XP professional SP-2 operating system (Microsoft Corp, Redmond, USA). After reconstruction, data were stored in individual DICOM files according to each patient.

## Cephalometric measurements

**Figure 1.**
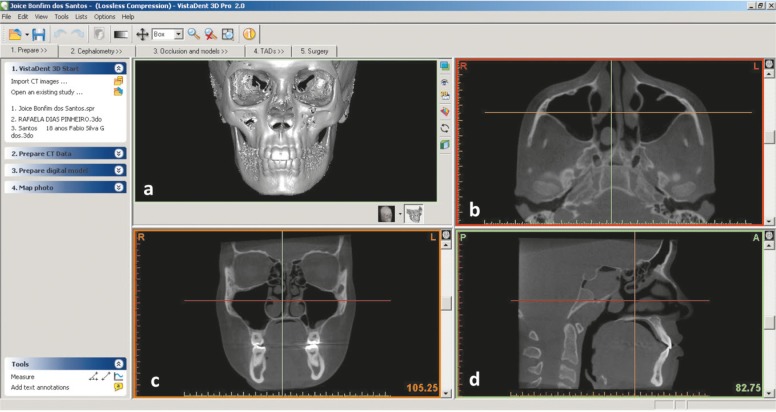
3D cephalometric module of VistaDent 3D Pro 2.00 software (Dentsply GAC, New York, USA). 3D reconstructions (A), Axial (B), coronal (C) and sagittal slices (D).

**Figure 2.**
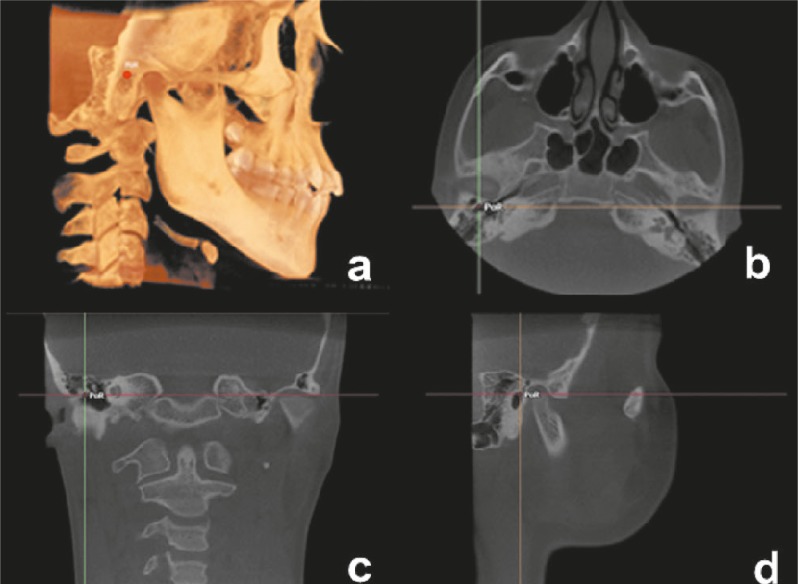
Right porion cephalometric landmark (PoR) identified in the 3D (A), axial (B), coronal (C) and sagittal (D) multiplanar reconstructions

After three-dimensional measurements were obtained, the DICOM files were imported into VistaDent 3D Pro 2.00 (Dentsply GAC, New York, USA). A total of 17 cephalometric landmarks selected according to a specific protocol for dentoskeletal symmetry assessment were identified by a calibrated operator, who had more than five years experience, and plotted by means of axial, coronal and sagittal multiplanar reconstruction (Table 1; Figs 1 and 2). Subsequently, reference planes were determined (Tables 2 and 3) and the linear measurements were automatically calculated by the software (Table 3; Figs 4 and 5). Values were recorded in a Microsoft Office Excel^(r)^ 2010 spreadsheet. Image upgrading and maximal magnification tools were used to ensure that all cephalometric landmarks were precisely plotted on each multiplanar reconstruction.

**Table 1.** Cephalometric landmarks

Cephalometric landmark	Cephalometric landmark description
Porion R (Po R)	The most superior point of the right auditory meatus
Porion L (Po L)	The most superior point of the left auditory meatus
Orbitale R (Or R)	The lowest point on the right inferior orbital margin
Orbitale L (Or L)	The lowest point on the left inferior orbital margin
Anterior nasal spine (ANS)	The lowest point of the maxillary anterior nasal spine
Posterior nasal spine (ENP)	The most posterior point of the maxillary posterior nasal spine
Capitulare R	Center of the head of right mandible
Capitulare L	Center of the head of left mandible
Condylion R (Co R)	The most superior posterior point of the right mandibular condyle
Condylion L (Co L)	The most superior posterior point of the left mandibular condyle
#16	The deepest point on the central fossa of right maxillary first molar
#26	The deepest point on the central fossa of left maxillary first molar
#36	Distobuccal cuspid tip of left mandibular first molar
#46	Distobuccal cuspid tip of right mandibular first molar
Gonion R (Go R)	The mid-point on the posterior outline of the angle of the mandible on the right side
Gonion L (Go L)	The mid-point on the posterior outline of the angle of the mandible on the left side
Gnathion (Gn)	The most anterior inferior point on the mandibular symphysis.

**Table 2.** Cephalometric measurements reference planes

Reference plane	Plane description
Frankfort horizontal plane (FHP)	Line connecting right and left porion to left orbitale
Coronal plane (CP)	Line connecting right and left porion, perpendicular to the Frankfort horizontal plane
Midsagittal plane (MSP)	Line connecting anterior and posterior nasal spines, perpendicular to the Frankfort horizontal plane
Maxillary horizontal plane (MHP)	Line connecting anterior and posterior nasal spines, perpendicular to the midsagittal plane
Mandibular plane (MP)	Line connecting right and left gonion to gnathion

**Table 3.** Cephalometric measurements

Maxilla	Description
#16 - Coronal plane	From #16 central fossa to coronal plane
#26 - Coronal plane	From #26 central fossa to coronal plane
#16 - Sagittal plane	From #16 central fossa to sagittal plane
#26 - Sagittal plane	From #26 central fossa to sagittal plane
#16 - ANS	From #16 central fossa to anterior nasal spine
#26 - ANS	From #26 central fossa to anterior nasal spine
#16 - Maxillary Plane Height	From #16 central fossa to maxillary horizontal plane
#26 - Maxillary Plane Height	From #26 central fossa to maxillary horizontal plane
#16 - FHP height	From #16 central fossa to Frankfort horizontal plane
#26 - FHP height	From #26 central fossa to Frankfort horizontal plane
Mandible	Description
#36 - Coronal plane	From #36 distobuccal cuspid to coronal plane
#46 - Coronal plane	From #46 distobuccal cuspid to coronal plane
#36-Gn	From #36 distobuccal cuspid to gnation
#46-Gn	From #46 distobuccal cuspid to gnation
#36 - Mandibular Plane Height	From #36 distobuccal cuspid to mandibular plane on the left side
#46 - Mandibular Plane Height	From #46 distobuccal cuspid to mandibular plane on the right side
Condylion R-Gn	From condylion to gnation
Condylion L-Gn	From condylion to gnation
Condylion R-GoR	From right condylion to right gonion
Condylion L-GoL	From left condylion to left gonion
Go R-Gn	From right gonion to gnation
Go L-Gn	From left gonion to gnation
FHP-Go R	From Frankfort horizontal plane to right gonion
FHP-Go L	From Frankfort horizontal plane to left gonion
TJD	Description
R Capitulare - sagittal plane	From R Capitulare to midsagittal plane
L Capitulare - sagittal plane	From L Capitulare to midsagittal plane
R Capitulare - coronal plane	From R Capitulare to coronal plane
L Capitulare - coronal plane	From L Capitulare to coronal plane
R Capitulare – FHP	From R Capitulare to Frankfort horizontal plane
L Capitulare – FHP	From L Capitulare to Frankfort horizontal plane

**Figure 3.**
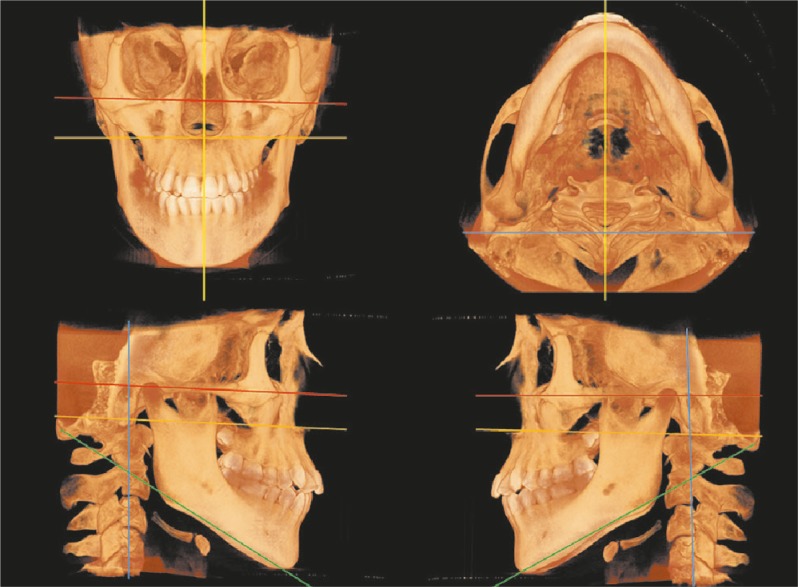
Three-dimensional reconstructions of the reference planes: Frankfort Horizontal Plane (red), Coronal Plane (blue), Midsagittal Plane (yellow), Maxillary Plane (orange) and Mandibular (green).

**Figure 4.**
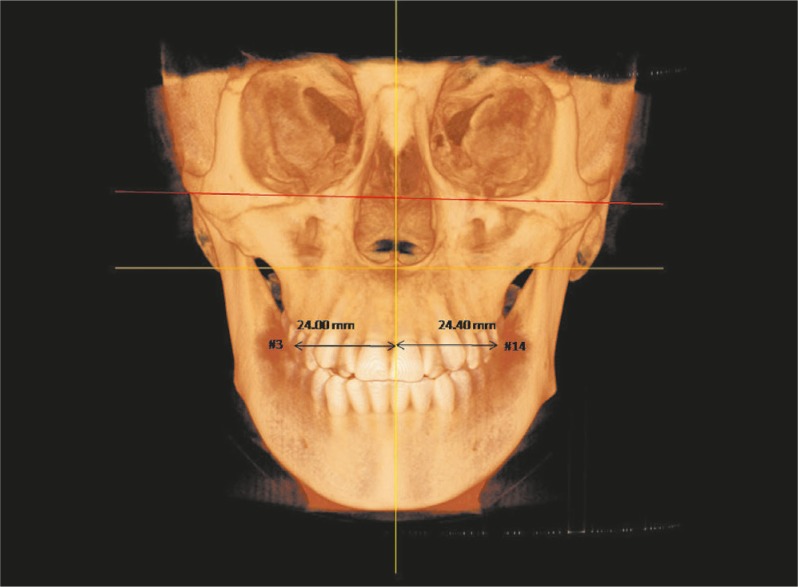
Three-dimensional image of cephalometric measurements between #16, #26 and the midsagittal plane.

**Figure 5.**
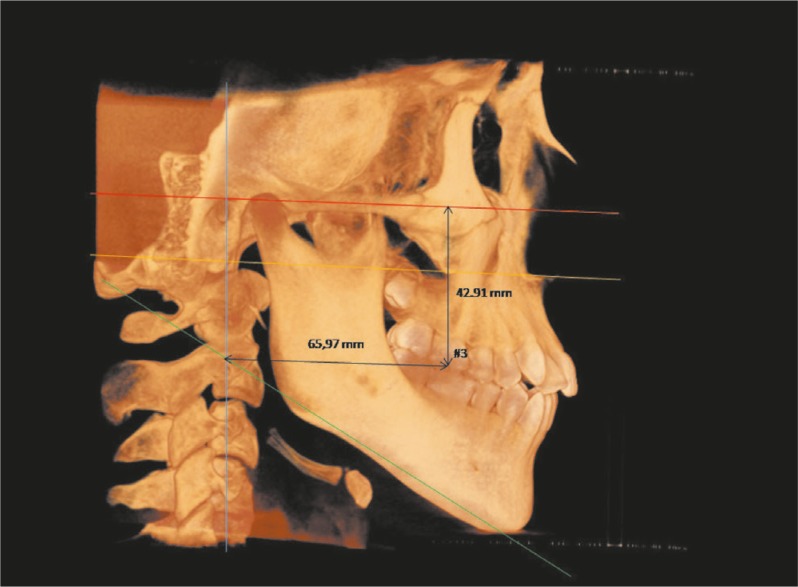
Three-dimensional image of cephalometric measurements from #16 landmark to the Frankfort Horizontal and Coronal Planes.

## Statistical analysis

Mean and standard deviation of all cephalometric measurements were obtained. Cephalometric measurements from both left and right sides and the differences between them were recorded in two subsequent tables. Those differences were assessed by t-test for paired samples and Wilcoxon test. Data normality was assessed by Kolmogorov-Smirnov test. Values were significant at P < 0.05.

Differences between measurements obtained on the left and right sides were recorded by descriptive statistics in Table 5 which shows minimal, maximum, mean and standard deviation values. All statistical analyses were performed by means of SPSS (20.0, SPSS Inc, Chicago, USA).

**Table 4.** Means and standard deviation of cephalometric measurements obtained from Angle Class I patients (n = 47).

Cephalometric measurements	Minimal and maximum values (mm)				Mean and standard deviation		
	Minimal	Maximum	Minimal	Maximum			
Maxilla	#16		#26		#16	#26	p
#16/26 - Coronal Plane	51.30	71.11	52.63	70.35	61.56 ± 4.47	61.22 ± 4.12	0.073
#16/26 - Sagittal Plane	19.96	26.47	19.56	26.51	23.33 ± 1.45	23.48 ± 1.51	0.453
#16/26 - ANS	38.60	51.74	38.36	51.37	44.75 ± 2.85	44.94 ± 2.91	0.240
#16/26 - MHP	15.10	25.69	14.61	27.44	20.56 ± 2.85	20.54 ± 2.79	0.348
#16/26 - FHP	31.97	49.21	31.32	47.82	40.36 ± 3.48	40.27 ± 3.45	0.610
Mandible	#46		#36		#36	#46	p
#16/26 - Coronal Plane	50.72	71.27	52.21	69.05	61.62 ± 4.33	61.60 ± 4.59	0.964
#16/26 - Gn	45.24	59.05	44.79	57.05	49.68 ± 2.50	49.74 ± 2.85	0.716
#16/26 - Height-GoGn	22.12	30.77	21.84	31.40	25.81 ± 2.19	25.92 ± 1.99	0.587
Mandible	Right		Left		Left	Right	p
Condylion-Gn	101.24	127.48	100.27	126.6	117.11 ± 4.74	117.42 ± 4.71	0.230
Condylion-Go	42.18	59.12	43.58	60.20	49.42 ± 3.33	49.84 ± 3.50	0.087
Go-Gn	76.45	92.85	77.61	90.4	84.51 ± 3.37	84.66 ± 3.44	0.569
FHP-Go	43.12	62.98	41.94	63.94	51.42 ± 4.23	51.88 ± 4.28	0.100
TMJ	Right		Left		Left	Right	p
Capitulare - MSP	43.83	51.43	42.55	51.25	47.84 ± 1.90	47.29 ± 2.17	0.036
Capitulare - Coronal Plane	6.66	12.96	5.93	13.18	10.18 ± 1.37	9.45 ± 1.32	0.000
Capitulare - FHP	3.27	11.61	3.40	11.65	7.33 ± 1.99	7.31 ± 1.79	0.894

**Table 5.** Means and standard deviation (SD) of differences between right and left sides in Angle Class I patients (n = 47).

Maxilla	Minimal	Maximum	SD
#16/26 - Coronal Plane	0.05	3.02	1.07 ± 0.76
#16/26 - Sagittal Plane	0.02	3.07	1.13 ± 0.68
#6 - ANS	0.06	2.19	0.93 ± 0.64
#6 - MHP	0.01	5.43	1.50 ± 1.38
#6 - FHP	0.05	2.52	0.87 ± 0.68
Mandible			
Coronal Plane	0.06	3.65	1.15 ± 0.81
Condylion - Gn	0	4.33	1.37 ± 1.11
Condylion - Go	0.01	4.01	1.38 ± 0.95
Go-Gn	0.02	4.53	1.38 ± 1.14
FHP - Go	0.01	5.35	1.55 ± 1.14
#6 - Gn	0.01	3.43	0.83 ± 0.77
Height - GoGn	0.05	5.33	0.98 ± 0.90
TMJ			
Capitulare - MSP	0.10	4.31	1.43 ± 1.13
Capitulare - Coronal Plane	0.04	2.45	0.90 ± 0.54
Capitulare - Frankfort	0.15	2.12	0.99 ± 0.56

## RESULTS

Results are summarized in Tables 4 and 5. Table 4 shows minimal, maximum, mean and standard deviation values of cephalometric measurements obtained from the maxilla, mandible and temporomandibular joint (TMJ). Table 5 shows the differences between left and right measurements.

## DISCUSSION

Facial harmony, an ancient esthetic concern of human beings, was confirmed by facial photographs of Angle Class I Brazilian patients, despite differences between right and left cephalometric measurements.

Orthodontic treatment is planned based on the linear and angular measurements of the craniofacial complex. For decades, measurements were taken on the basis of two-dimensional images. Lateral and posteroanterior cephalograms as well as panoramic radiographs were often used as complementary examination by specialized dentists, mainly in Orthodontics.^26^^,^ 31 ^-^ 38 Measurements are usually obtained on the basis of two-dimensional scans of three-dimensional structures.

CBCT has redefined cephalometric analysis.^27^^-^ 30 ^,^ 39 Methods may have to be adapted to CBCT risks and benefits, as well as to its three-dimensional scans so as to increase the accuracy of cephalometric measurements.

This study used VistaDent 3D Pro 2.00 (Dentsply GAC, Nova York, USA) which enables navigation in the axial, sagittal and coronal planes so as to take cephalometric measurements. Measurements taken on the basis of CBCT scans are more accurate and reliable due to better magnification and less distortion than two-dimensional images.^26^^,^ 27 ^,^ 40 ^-^ 43

Three-dimensional cephalometric analyses were carried out to establish reference values. Sievers et al.^44^ assessed 70 patients and used the index by Katsumata et al^24^ to measure asymmetry in Class I and II patients. The index was calculated based on the distances from the craniometric landmarks to the midsagittal, coronal and axial planes. The midsagittal plane was established by sella, nasio and dent landmarks; whereas the axial plane was established by the sella and nasio landmarks and was perpendicular to the midsagittal plane. Dent landmark was used to determine the coronal plane which was perpendicular to the other two planes. Angle Class II patients were not more asymmetrical than Class I patients.

In this study, landmarks and measurements were used to assess symmetry according to five planes: midsagittal, coronal, Frankfort horizontal, maxillary and mandibular. These planes were used as reference for cephalometric measurements. The midsagittal plane was established by the anterior and posterior nasal spines and was perpendicular to the Frankfort horizontal plane according to a model, which differs Katsumata et al.^24^ The coronal plane connected the right and left porion and was perpendicular to the Frankfort horizontal plane. There were significant differences in Capitulare-MSP and Capitulare Coronal-Plane cephalometric measurements.

Using different methods to locate craniometric landmarks and three-dimensional cephalometric measurements affects the process of establishing reference values, which hinders comparison with results yielded by previous studies.^24^^,^ 27 ^,^ 44 ^,^ 45 Some studies have used algorithms to demonstrate the use of three-dimensional cephalometry and to derive two-dimensional cephalometric references for three-dimensional evaluations.^26^^,^ 41 ^,^ 46 New cephalometric methods using three-dimensional scans have been suggested.^27^^,^ 28 ^,^ 29 Cheung et al^29^ developed a model of cephalometric analysis of dentofacial abnormalities and established new cephalometric reference values for Chinese adult patients. Cavalcanti et al^30^ assessed the accuracy of craniofacial bone and tissue measurements obtained by means of 3D computed tomography (CT) and a volume technique using an independent workstation with graphic tools. The 3D-CT measurements proved accurate in assessing growth and developmental changes. Takahashi et al^3^ assessed facial skeletal structures using the vertical view of cephalometric lateral radiographs not only to establish the mean normality values for young Brazilians whose ancestors were white or Asian with normal occlusion, but also to assess the differences between males and females and ethnic groups under study. Their results suggested that males and females from both ethnic groups presented differences in some of the cephalometric measurements. Additionally, differences between the two ethnic groups under study were also observed.

The reference values obtained in this study are complementary to other dentoskeletal symmetry findings, such as those provided by clinical and model analyses. Tooth size discrepancies may result in midline deviation which also leads to asymmetry. The Bolton discrepancy analysis of digital CBCT models has been used to assess the effect of teeth on asymmetry. Tarazona et al^47^ assessed the reproducibility and reliability of the Bolton index when using digital CBCT models and digitized images of conventional models. Although both methods proved clinically acceptable, CBCT results were accurate and reproducible. Sanders et al^48^ compared the degree of dentoskeletal asymmetry in Class II patients and subjects with normal occlusion by means of CBCT. A total of 34 landmarks were used to assess dental, dentoalveolar, bone and condyle asymmetries. The distances from the contact points of maxillary and mandibular central incisors to the midsagittal plane were measured together with linear and angular measurements so as to establish dentoskeletal asymmetry. These measurements were essential for the precise diagnosis of dentoskeletal symmetry.

Asymmetries may result in esthetic and functional deviations of variable intensity. Thus, using cephalometry to determine the severity of asymmetry is an essential tool in orthodontic planning. CBCT may be used for cephalometric analysis, but this three-dimensional tool exposes patients to radiation. Therefore, care should be taken to ensure the best cost-benefit relationship between information and radiation dose,^22^^,^ 23 and decisions should respect the ALARA principle (as-low-as-reasonably-achievable).

Further studies should be conducted to determine the clinical significance of differences and standard deviations. The faces of subjects included in our study were symmetrical, but cephalometric measurements revealed differences between the left and right sides as well as statistical differences in two cephalometric measurements of TMJ. Despite this discrepancy, CBCT scans may function as a three-dimensional guide to identify and measure dentoskeletal asymmetries during orthodontic and surgical planning.

## CONCLUSION

The faces of Angle Class I subjects included in our study were symmetrical, but cephalometric measurements revealed differences between the left and right sides.

## References

[1] Broadbent HB (1931). A new x-ray technique and its application to
orthodontia. Angle Orthod.

[2] Graber TM (1966). Orthodontics principles and practice.

[3] Takahashi R, Pinzan A, Henriques JFC, Freitas MR, Janson GRP, Almeida RR (2005). Análise cefalométrica comparativa das alturas faciais,
anterior e posterior, em jovens brasileiros, descendentes de xantodermas e
leucodermas, com oclusão normal. Rev Dental Press Ortod Ortop Facial.

[4] Kau CH, Bozic M, English J, Lee R, Bussa H, Ellis RK (2009). Cone-beam computed tomography of the maxillofacial
region: an update. Int J Med Robotics Comput Assist Surg.

[5] Farman AG, Scarfe WC (2006). Development of imaging selection criteria and procedures
should precede cephalometric assessment with cone-beam computed
tomography. Am J Orthod Dentofacial Orthop.

[6] Castro IO, Alencar AH, Valladares-Neto J, Estrela C (2013). Apical root resorption due to orthodontic treatment
detected by cone beam computed tomography. Angle Orthod.

[7] Angle EH (1899). Classification of malocclusion. Dent Cosmos.

[8] Arai Y, Tammisalo E, Hashimoto K, Shinoda K (1999). Development of a compact computed apparatus for dental
use. Dentomaxillfac Radiol.

[9] Estrela C, Bueno MR, Alencar AHG, Mattar R, Valladares-Neto J, Azevedo BC (2009). Method to evaluate inflammatory root resorption by using
cone beam computed tomography. J Endod.

[10] Dreiseidler T, Mischkowski RA, Neugebauer J, Ritter L, Zöller JE (2009). Comparison of Cone-Beam Imaging with orthopantomography
and computerized tomography for assessment in presurgical implant
dentistry. Int J Oral Maxillofac Implants.

[11] Lund H, Gröndahl K, Gröndahl H (2010). Cone beam computed tomography for assessment of root
length and marginal bone level during orthodontic treatment. Angle Orthod.

[12] Kau CH, Richmond S, Palomo JM, Hans MG (2005). Three-dimensional cone beam computerized tomography in
orthodontics. J Orthod.

[13] Mozzo P, Procacci C, Tacconi A, Martini PT, Andreis IA (1998). A new volumetric CT machine for dental imaging based on
the cone-beam technique: preliminary results. Eur Radiol.

[14] Capelozza L, Fattori L, Matagliatti L (2005). Um novo método para avaliar as inclinações dentárias
utilizando a tomografia computadorizada. Rev Dental Press Ortod Ortop Facial.

[15] Cattaneo PM, Bloch CB, Calmar D, Hjortshøj M, Melsen B (2008). Comparison between conventional and cone-beam computed
tomography-generated cephalograms. Am J Orthod Dentofacial Orthop.

[16] Farman AG, Scarfe WC (2006). Development of imaging selection criteria and procedures
should precede cephalometric assessment with cone-beam computed
tomography. Am J Orthod Dentofacial Orthop.

[17] Oliveira AE, Cevidanes LH, Phillips C, Motta A, Burke B, Tyndall D (2009). Observer reliability of three-dimensional cephalometric
landmark identification on cone-beam computerized tomography. Oral Surg Oral Med Oral Pathol Oral Radiol Endod.

[18] Cavalcanti MGP, Sales MAO, Cavalcanti MGP (2008). Tomografia computadorizada. Diagnostico por Imagem da Face.

[19] Dudic A, Giannopoulou C, Leuzinger M, Kiliaridis S (2009). Detection of apical root resorption after orthodontic
treatment by using panoramic radiography and cone-beam computed tomography of
super-high resolution. Am J Orthod Dentofacial Orthop.

[20] You KH, Lee KJ, Lee SH, Baik HS (2010). Three-dimensional computed tomography analysis of
mandibular morphology in patients with facial asymmetry and mandibular
prognathism. Am J Orthod Dentofacial Orthop..

[21] Freitas JC, Alencar AHG, Estrela C (2013). Long-term evaluation of apical root resorption after
orthodontic treatment using periapical radiography and cone beam computed
tomography. Dental Press J Orthod.

[22] Garcia Silva MA, Wolf U, Heinicke F, Gründler K, Visser H, Hirsch E. M. (2008). Effective dosages for recording Veraviewepocs dental
panoramic images: analog film, digital, and panoramic scout for
CBCT. Oral Surg Oral Med Oral Pathol Oral Radiol Endod.

[23] Silva MA, Wolf U, Heinicke F, Bumann A, Visser H, Hirsch E (2008). Cone-beam computed tomography for routine orthodontic
treatment planning: a radiation dose evaluation. Am J Orthod Dentofacial Orthop.

[24] Katsumata A, Fujishita M, Maeda M, Ariji Y, Ariji E, Langlais RP (2005). 3D-CT evaluation of facial asymmetry. Oral Surg Oral Med Oral Pathol Oral Radiol Endod.

[25] Gribel BF, Gribel MN, Frazäo DC, McNamara JA, Manzi FR (2011). Accuracy and reliability of craniometric measurements on
lateral cephalometry and 3D measurements on CBCT scans. Angle Orthod.

[26] Gribel BF, Gribel MN, Manzi FR, Brooks SL, McNamara JA (2011). From 2D to 3D: an algorithm to derive normal values for
3-dimensional computerized assessment. Angle Orthod.

[27] Swennen GR, Schutyser F, Barth EL, De Groeve P, De Mey A (2006). A new method of 3-D cephalometry part I: The anatomic
Cartesian 3-D reference system. J Craniofac Surg.

[28] Swennen GR, Schutyser F (2006). Three-dimensional cephalometry: spiral multislicevs
cone-beam computed tomography. Am J Orthod Dentofacial Orthop.

[29] Cheung LK, Chan YM, Jayaratne YS, Lo J (2011). Three-dimensional cephalometric norms of Chinese adults
in Hong Kong with balanced facial profile. Oral Surg Oral Med Oral Pathol Oral Radiol Endod.

[30] Cavalcanti M, Rocha S, Vannier MW (2004). Craniofacial measurements based on 3D-CT volume
rendering: implications for clinical applications. Dentomaxillofac Radiol.

[31] Chidiac JJ, Shofer FS, Al-Kutoub A, Laster LL, Ghafari J (2002). Comparison of CT scanograms and cephalometric
radiographs in craniofacial imaging. Orthod Craniofac Res.

[32] Cevidanes LHS, Styner MA, Profit WR (2006). Image analysis and superimposition of 3-Dimensional
cone-beam computed tomography models. Am J Orthod Dentofacial Orthop.

[33] Moraes ME, Hollender LG, Chen CS, Moraes LC, Balducci I (2011). Evaluating craniofacial asymmetry with digital
cephalometric images and cone-beam computed tomography. Am J Orthod Dentofacial Orthop.

[34] Janson GR, Metaxas A, Woodside DG, Freitas MR, Pinzan A (2001). Threedimensional evaluation of skeletal and dental
asymmetries in Class II subdivision malocclusions. Am J Orthod Dentofacial Orthop.

[35] Rose JM, Sadowsky C, BeGole EA, Moles R (1994). Mandibular skeletal and dental asymmetry in Class II
subdivision malocclusions. Am J Orthod Dentofacial Orthop.

[36] Bruntz LQ, Palomo JM, Baden S, Hans MG (2006). A comparison of scanned lateral cephalograms with
corresponding original radiographs. Am J Orthod Dentofacial Orthop.

[37] Isik F, Nalbantgil D, Sayinsu K, Arun T (2006). A comparative study of cephalometric and arch width
characteristics of Class II division 1 and division 2
malocclusions. Eur J Orthod.

[38] Sayinsu K, Isik F, Trakyali G, Arun T (2007). An evaluation of the errors in cephalometric
measurements on scanned cephalometric images and conventional
tracings. Eur J Orthod.

[39] Hajeer MY, Millett DT, Ayoub AF, Siebert JP (2004). Applications of 3D imaging in orthodontics: part
I. J Orthod.

[40] Adams GL, Gansky SA, Miller AJ, Harrell WE, Hatcher DC (2004). Comparison between traditional 2-dimensional
cephalometry and a 3-dimensional approach on human dry skulls. Am J Orthod Dentofacial Orthop.

[41] Cho Y, Moseley DJ, Siewerdsen JH, Jaffray DA (2005). Accurate technique for complete geometric calibration of
cone-beam computed tomography systems. Med Phys.

[42] Hilgers ML, Scarfe WC, Scheetz JP, Farman AG (2005). Accuracy of linear temporomandibular joint measurements
with cone-beam computed tomography and digital cephalometric
radiography. Am J Orthod Dentofacial Orthop.

[43] Santoro M, Jarjoura K, Cangialosi TJ (2006). Accuracy of digital and analogue cephalometric
measurements assessed with the sandwich technique. Am J Orthod Dentofacial Orthop.

[44] Sievers MM, Larson BE, Gaillard PR, Wey A (2012). Asymmetry assessment using cone beam CT A Class I and
Class II patient comparison. Angle Orthod.

[45] Peck JL, Sameshima GT, Miller A, Worth P, Hatcher DC (2007). Mesiodistal root angulation using panoramic and Cone
Beam CT. Angle Orthod.

[46] Halazonetis DJ (2005). From 2-dimensional cephalograms to 3-dimensional
computed tomography scans. Am J Orthod Dentofacial Orthop.

[47] Tarazona B, Llamas JM, Cibrián R, Gandía JL, Paredes V (2012). Evaluation of the validity of the Bolton Index using
cone-beam computed tomography (CBCT). Med Oral Patol Oral Cir Bucal.

[48] Sanders DA, Rigali PH, Neace WP, Uribe F, Nanda R (2010). Skeletal and dental asymmetries in Class II subdivision
malocclusions using cone-beam computed tomography. Am J Orthod Dentofacial Orthop..

